# Paper on Quarantine

**Published:** 1879-05

**Authors:** 


### Editorial.

---

It will be remembered by those in attendance at the last meeting of the Medical Association of Georgia, that the paper of Dr. J. C. LeHardy, on quarantine, was incomplete in the statistical account of expenses necessary to support quarantine. In the reading of his paper this fact was made known, and the failure to receive up to that time some promised facts connected therewith, from a certain reliable source.

The enormous amount of money required for this purpose, as shown from the data then at his command, would seem to be sufficient to, at least, stir up national legislators to the importance of becoming satisfied that quarantine will prevent the introduction of disease, before they authorize so monstrous a measure as is contemplated.

Dr. LeHardy received the expected statistics on his arrival home, as follows:

FORT LEE, April 14th, 1879.

DR. J. C. LEHARDY, Savannah, Ga.

*Dear Doctor:*—On my return from the city (N. Y.) on Friday, I that night received your postal card and I again wrote Dr. Vanderpoel. The party who was to get for me the number of vessels arriving in New York harbor during the year, was detained on board steamers in his official capacity, and did not get to the custom house (office) books till Monday (to-day). He gave me to-night the following list of foreign (vessels) arrivals during 1878, and up to this month. He says he did not understand me that I wanted also a list of "Coasters," but will get the number tomorrow for me, but says that the number of coasters will be near about as many, if not more than the subjoined list of *foreign* vessels. 1878.—1,310 steamers; 540 ships; 2,949 barks; 1,021 brigs; 1,528 schooners. 1879.—January.—114 steamers; 37 ships; 221 barks; 77 brigs; 98 schooners. February.—107 steamers; 33 ships; 152 barks; 61 brigs; 66 schooners. March.—113 steamers; 69 ships; 260 barks; 94 brigs; 118 schooners.

I wrote you the relative fees demandable by Health Officer (Dr. Vanderpoel) for examination, and the fumigation fee in addition, and in my card to you I explained that these were *demandable* fees, but that the probabilities were that *often more* was paid and received. I also explained that Dr. Vanderpoel's desire certainly was that no one should know what his position was worth, nor was I aware that the statute requires him (even to the State Department at Albany) to make any itemized report. I am positively assured that no report by him is made, either to the Municipal Head-quarters or to the U. S. Custom House; his office being a gift from the government at Albany. The fees are different in different (State) ports, and the position (office) is of value in proportion to the amount of commerce. Dr. Vanderpoel, in conversation with me, said you can tell your friend that "he will be safe in saying that the entry fees of New York are cheaper than those of any other port, and that an in-coming vessel is quarantined with less incommode than at any other port in America.

Now, as to the working of the quarantine, I think, perhaps, you may think the following abstract from his articles on quarantine an *apropos* confession :

Page 22. "I am fortified by a long experience and by the fact that from the worst infected vessels no unpleasant consequences have followed where the simple process of æration, *cleaning*, fumigation and disinfection have been thorough. What I have said with regard to fumigation also applies to disinfection. No *elaborate* or expensive machine, no high-priced or secret compounds are needed. A solution of sulphate of iron, one pound to the gallon of water, combined with carbolic acid of twenty per cent. strength thoroughly applied with a common watering pot, will meet every contingency." Again: "Such preparations and machines are worse than useless, for not only are they inefficacious for the purposes intended, but they *beget a false confidence*, which causes the neglect of that most important of all disinfectants, *absolute cleanliness*. Disinfectants, fumigation, and all that class of measures should be regarded merely as *adjuvants*, useful for the purpose of reaching portions of the vessel which, in the process of cleaning, may *possibly* have been overlooked, or when the cleansing could not have been absolutely thorough. I am not aware that chemistry presents any two articles more readily applied, more rapid of generation, or possessed of greater oxidizing and permeating properties than *sulphurous acid gas* and *chlorine*."

There is one other important quotation, interpret it as an *honest* conviction or one guided by the pocket, just as you please: "The longer such a vessel is allowed to remain untouched—i. e. the longer a quarantine is exacted—the greater will be the virulence of the disease and the greater the danger of spreading the infection." Then, if you are so liable to have *too much of a good thing*, wherein is the *germ* of its goodness, except in the fees?

I guess I have scribbled enough. He says that the captains say that if, when in a port, (yellow fever) they daily pump out the bilge, and pump in other water and out again, they are then insured from yellow fever.

(Signed) A. Clendinen.

With such facilities given to vessels coming from infected ports, is it astonishing that our ports should be deserted, where the detention at quarantine extends from ten to twenty days? J. LeH.

